# Survival of Microorganisms on Nonwovens Used for the Construction of Filtering Facepiece Respirators

**DOI:** 10.3390/ijerph16071154

**Published:** 2019-03-31

**Authors:** Katarzyna Majchrzycka, Małgorzata Okrasa, Justyna Szulc, Anita Jachowicz, Beata Gutarowska

**Affiliations:** 1 Department of Personal Protective Equipment, Central Institute for Labour Protection—National Research Institute, Wierzbowa 48, 90-133 Łódź, Poland; kamaj@ciop.lodz.pl; 2 Institute of Fermentation Technology and Microbiology, Faculty Biotechnology and Food Science, Lodz University of Technology, Wólczańska 171/173, 90-924 Łódź, Poland; justyna.szulc@p.lodz.pl (J.S.); 801208@edu.p.lodz.pl (A.J.); beata.gutarowska@p.lodz.pl (B.G.)

**Keywords:** filtering nonwovens, microorganisms survivability, filtering facepiece respirators, respiratory protection

## Abstract

Filtering nonwovens that constitute the base material for filtering facepiece respirators (FFRs) used for the protection of the respiratory system against bioaerosols may, in favourable conditions, promote the development of harmful microorganisms. There are no studies looking at the impact that different types of filtering nonwovens have on microorganism survival, which is an important issue for FFR producers and users. Five commercial filtering nonwovens manufactured using diverse textile technologies (i.e., needle-punching, melt-blown, spun-bonding) with different structural parameters and raw material compositions were used within our research. The survival of microorganisms on filtering nonwovens was determined for *E. coli*, *S. aureus*, *B. subtilis* bacteria; *C. albicans* yeast and *A. niger* mould. Samples of nonwovens were collected immediately after inoculum application (at 0 h) and after 4, 8, 24, 48, 72, and 96 h of incubation. The tests were carried out in accordance with the AATCC 100-1998 method. Survival depended strongly on microorganism species. *E. coli* and *S. aureus* bacteria grew the most on all nonwovens tested. The structural parameters of the nonwovens tested (mass per unit area and thickness) and contact angle did not significantly affect microorganism survival.

## 1. Introduction

The ability to prevent the harmful impact of biological agents on humans depends largely on the efficacy and quality of the protection method or equipment used. Given that a significant fraction of pathogenic microorganisms is transmitted by dust or droplets, filtering facepiece respirators (FFRs), whose base material are filtering nonwovens, are an important method of protection. The use of respiratory protecting equipment of this type is common in many workplaces. The deployment of FFRs for work-unrelated applications is also becoming more common [1,2]. This is due to the rapidly spreading epidemics of influenza and other infectious diseases, as well as due to an increased public awareness concerning environmental threats.

Protection against biological agents is particularly important in health care, where the use of surgical respirators (SRs), which are manufactured from filtering nonwovens, is common. SRs have been used for over 100 years to protect patients from droplet-transmitted infections. Moreover, SRs are frequently perceived as personal protection equipment for medical personnel, although their protective efficacy is at a relatively low level compared to standard FFRs.

Filtering facepiece respirators have to comply with European standard requirements as a product ensuring human safety [3]. Unfortunately, the standards do not define requirements or research methods that would consider the specifics of biological factors. In particular, there are no standards for the assessment of microorganism survival rate on filtering material. Therefore, despite numerous research and development studies aimed at conferring biocidal properties to filtering nonwovens [4,5,6,7,8,9,10,11,12,13,14,15], there are no FFRs on the market that guarantee restricted growth of microorganisms accumulated on the filtering material during use. At the same time, many studies [16,17,18,19,20,21,22,23] confirmed that during prolonged FFR use against bioaerosols, bacterial numbers may increase, and biofilms may form on the filtering nonwoven, which may become a potential threat for the user. Likewise, it was found that during influenza A (H1N1) pandemics, viruses and microorganisms survived on the filtering material of SRs used by the personnel for several hours to several days [24,25,26]. Therefore, the knowledge of factors affecting dynamics of microorganism growth on filtering materials is particularly important to the FFR manufacturer and user.

Moisture and mineral dust from work environments may promote microorganism growth within filtering nonwovens [21,22,23]. Based on breathing simulation, Majchrzycka et al confirmed that moisture from exhaled air, which accumulate within FFRs creates favourable conditions for microorganism growth [21]. Such conditions persist over time when FFRs are in use, even if there are breaks. Environmental conditions have a significant impact on the survival of some microorganism species on nonwovens. There is a dearth of research concerning the influence of the type of filtering nonwovens on this phenomenon.

Filtering facepiece respirators are multilayer products. They consist of a thin layer of a protective nonwoven on both sides, i.e., both on the side exposed to bioaerosol influx from the working environment (external part of the equipment) and the one in contact with the user’s face (inside of the equipment). Its mass per unit area is usually between 20 and 50 g/m^2^. The next layer is a needled nonwoven responsible for pre-filtration. Its mass per unit area can be as high as 250 g/m^2^. It is often subjected to a high temperature calendering process that thickens the nonwoven structure and gives it rigidity. This helps form the appropriate facepiece shape and ensures that it does not change over time when the FFR is in use. The high efficiency melt-blown electret nonwoven (formed by blowing the melted polymer) constitutes the most important layer. It is responsible for the efficiency of filtration. Due to this function, it is usually an electret nonwoven (electrified by corona discharge). Nonwovens made of polypropylene (PP) are widely used in FFRs; albeit those made of polyacrylonitrile (PAN) or polyethylene (PE) also exist.

Mass per unit area, thickness, porosity, and diameter of elementary fibres are the basic parameters determining the properties of the nonwovens used for FFR construction. All of these can contribute to the survival of microorganisms on the filtering material. The hydrophobicity of the elementary fibre is another factor that may affect microorganism survival. Therefore, the aim of this study was to assess the survival of selected bacteria, yeasts, and moulds (*E. coli*, *S. aureus*, *B. subtilis*, *C. albicans* and *A. niger*) on five types of filtering nonwovens most often used for FFR construction over a duration corresponding to the time of use on consecutive working days. In addition, the article discusses a correlation between microorganism survival and the mass per unit area and thickness of the nonwoven and its contact angle.

## 2. Materials and Methods

### 2.1. Filtering Nonwovens

Five filtering nonwovens typically used for FFR construction described in Table 1 were used within the study.

### 2.2. Assessment of the Survival of Microorganisms on Nonwovens

The microorganisms stored in the Pure Culture Collection ŁOCK 105 were used to study survival on filtering nonwovens (Table 2). The selected microorganisms belonged to various taxonomic groups (bacteria, yeasts, moulds) and were characterized by diverse physiology of growth.

Inocula of bacteria and yeast were prepared by inoculating 20 ml of sterile tryptic soy broth (TSB, Merck, Kenilworth, NJ, USA) or malt extract broth (MEB, Merck) medium for bacteria or yeast, respectively. Inoculum suspensions were incubated at 30 ± 2 °C for 24 h. Mould inoculum was obtained by washing the spores of malt extract agar (MEA, Merck) slants containing 7-day *A. niger* cultures using MEB medium. In this way, microorganism suspensions of 3.79 × 10^7^–1.12 × 10^9^ CFU/mL density were obtained (Table 2). Then, 25 µL of the suspension inocula were applied homogenously onto the UV-disinfected nonwoven swatches of 4 cm^2^ (2 × 2 cm squares) surface area using pipette with sterile tips for the distribution of small water droplets over the whole samples. The swatches were then placed in sterile Petri dishes and incubated in Binder-720 climatic chamber at 28 ± 2 °C and relative humidity of 80%. 

Quantitative static AATCC 100-1998 “Antimicrobial Finishes of Textile Materials” method was used to determine microorganism survival on the nonwovens [27]. Test samples were taken immediately after inoculum application (time 0h) and after 4, 8, 24, 48, 72 and 96 h of incubation. The test was performed according to methodology described in [21].

Nine results obtained in Grubbs test were selected for statistical analysis, after discarding uncertain results. Arithmetic mean and standard deviation of the number of microorganisms grown on the plates over consecutive hours of the experiment were calculated using Microsoft® Excel. In addition, statistical differences between microorganism number at t = 0 and after 4, 8, 24, 48, 72 and 96 h, and between the number of microorganisms over consecutive incubation hours were calculated (one-way analysis of variance (ANOVA), significance level α = 0.05). The normality was checked with Shapiro-Wilk test, which was followed by Levene’s test to assess the equality of variances. Statistically significant differences in microorganism number on different types of nonwovens incubated for the same length of time (one- and two-way ANOVA, significance level α = 0.05) were determined using the STATISTICA 13.1 software (StatSoft, Kraków, Poland). Ultimately, the results were compared using Tukey test (significance level α = 0.05). Microorganism survivability (N) was calculated after 4, 8, 24, 48, and 96 h of incubation as a ratio of the number of microorganisms after a given incubation time to the initial number of microorganisms.

### 2.3. Structural Parameters and Contact angle Determination

The mass per unit area of nonwovens was determined according to methodology described in EN 29073-1:1992 standard [28] using R160P laboratory balance (Sartorius GMBH, Goettingen, Germany). Their thickness was ascertained according to ISO 5084:1996 [29] using the J-40-T digital material thickness gauge (CheckLine, Bad Bentheim, Germany). The contact angle was determined using the Phoenic-Alpha contact angle apparatus (SEO, Suwon-si, Republic of Korea) equipped with an attachment for measuring contact angle at low and high temperatures. During the test, a drop of liquid was dispensed onto the sample surface using a syringe with a needle. The image of the drop was recorded using a high-resolution camera and then exported to ImageJ software for contact angle measurement.

## 3. Results and Discussion

### 3.1. Assessment of the Survival of Microorganisms on Nonwovens

**Figure 1:**
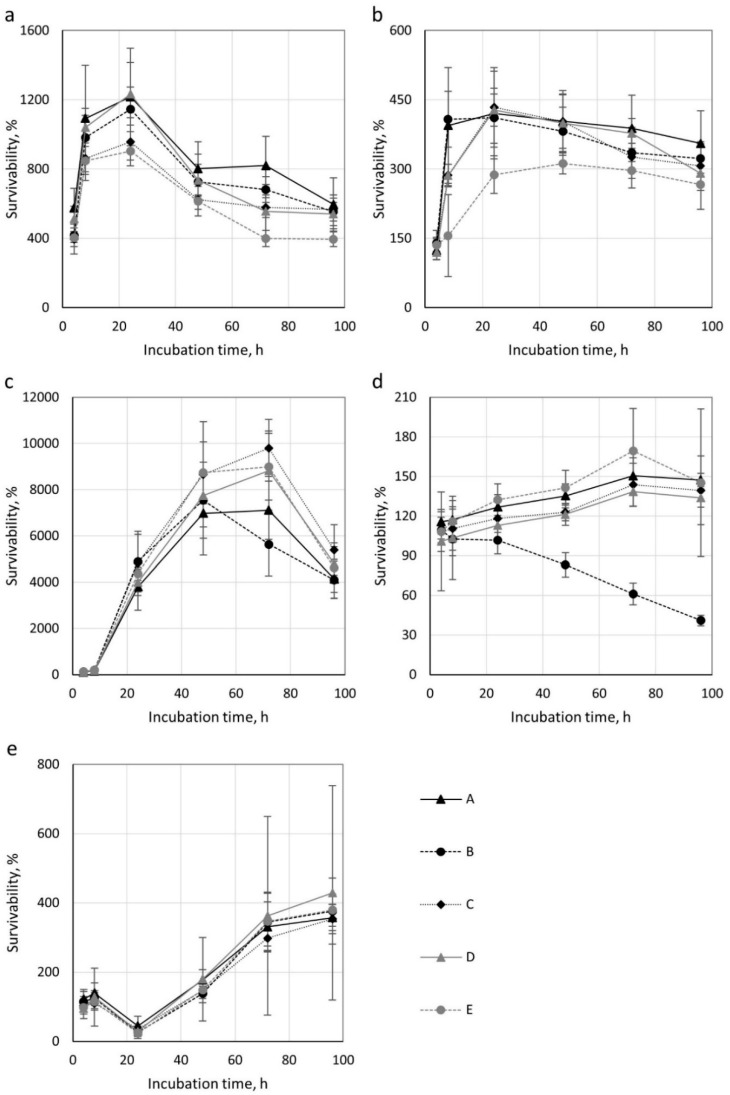
Survivability of microorganisms on nonwovens depending on incubation time; (**a**) *E. coli*, (**b**) *S. aureus*, (**c**) *B. subtilis*, (**d**) *C. albicans*, (**e**) *A. niger*.

The number of microorganisms on the filtering nonwovens studied at 0, 4, 8, 24, 48 and 96 h of incubation is shown in Table S1, and microorganism survival at subsequent time points in Figure 1.

The number of *E. coli* bacteria on the nonwovens varied 5.07 × 10^5^–7.54 × 10^6^ CFU/sample depending on the nonwoven type and incubation time. Statistically significant differences were observed in the number of *E. coli* bacteria between the control sample (t = 0 h) and each subsequent incubation time for all nonwovens studied. Statistically significant differences were also seen in the number of *E. coli* bacteria on nonwovens A, B and E at incubation times of 8, 24, 72 and 96 h compared to the remaining samples.

The number of *S. aureus* bacteria on the nonwovens equalled 5.33 × 10^5^–2.45 × 10^6^ CFU/sample depending on the nonwoven type and incubation time. Statistically significant differences were observed in the number of *S. aureus* bacteria between the control sample (t = 0 h) and each subsequent time point for all nonwovens studied except for nonwovens A and D at 4 h of incubation. When comparing bacterial numbers for consecutive incubation times, statistically significant differences were observed for nonwovens A and E and nonwoven B following 8 h; nonwoven E and other nonwovens tested after 24 h; and for nonwovens E and A after 96 h incubation.

The number of *B. subtilis* bacteria on the nonwovens studied equalled 1.47 × 10^4^–1.43 × 10^6^ CFU/sample depending on the nonwoven type and incubation time, depending on the incubation time. Statistically significant differences were observed in bacterial numbers between the control sample (t = 0 h) and after 8, 24, 58, 72 and 96 h of incubation for each nonwoven studied. When comparing bacterial numbers for consecutive incubation times, statistically significant differences were observed for nonwoven B and other nonwovens following 72 h of incubation, and for nonwovens B and D following 96 h incubation.

The number of *C. albicans* yeast on the studied nonwovens was in the range 3.02 × 10^4^–5.49 × 10^4^ CFU/sample depending on the nonwoven type and the incubation time. In all nonwovens, the number of *C. albicans* increased with the length of incubation time. An exception was nonwoven B, where a gradual decrease was seen. Such changes were not always statistically significant. When comparing yeast numbers on nonwoven types for consecutive incubation times, statistically significant differences were observed between nonwovens A and B, and between nonwovens B and C following 24 h of incubation. In addition, statistically significant differences were also observed between nonwoven B and all the others following 48, 72, and 96 h of incubation.

The number of *A. niger* mould on the studied nonwovens equalled 5.00 × 10^3^–6.62 × 10^4^ CFU/sample depending on the nonwoven type and incubation time depending on the incubation time. In all nonwovens, the number of *A. niger* was the lowest after 24 h of incubation and subsequently increased, reaching a peak after 96 h of incubation. No statistically significant differences in mould numbers were detected for consecutive incubation times for the various types of nonwovens.

*E. coli* survival rate on the nonwovens ranged from 394 to 1234%. This index reached the highest values (904–1234%) after 24 h and the lowest (394–595%) after 4 and 96h of incubation. The survival rate of *S. aureus* bacteria was lower and between 119–434%. Depending on the type of nonwoven, the index reached the highest values (156–434%) following 8, 24, and 48 h of incubation. The lowest survival rate (119–139%) of *S. aureus* was recorded after 4h of incubation. The survival rate of *B. subtilis* was 105–9804%. The index reached the highest value (5637–9804%) after 72 h except for nonwoven B, for which the highest survival was documented after 48h of incubation (7539%). The lowest *B. subtilis* survival was recorded after 4 h of incubation for all nonwovens tested. For *C. albicans* yeast, the survival rate was in the range of 41–169%. This index reached the highest value (138–169%) after 72 h of incubation except for nonwoven B, for which the highest survival rate (109%) was found after 4 h. The lowest survival rate for *C. albicans* (101%–116%) was recorded after 4h incubation for nonwovens A, C, D and E and after 96h (41%) for nonwoven B. The survival rate of *A. niger* mould was 25–429%. The index reached the highest values (354–429%) after 96 h incubation, and the lowest (25–45%) after 24 h.

The survival of microorganisms on the nonwovens tested was the highest in the case of bacteria *B. subtilis* (105–9804%), *E. coli* (394–1234%) and *S. aureus* (119–439%). It was lower for of *A. niger* mould (25–429%) and lowest for *C. albicans* yeast (41–169%). The maximal survival rate was achieved the quickest (at 24h of incubation) by *E. coli* (904–1234%). *S. aureus* bacteria achieved maximal survival rate very quickly, as well (312–434% after 8, 24 and 48 h depending on the nonwoven). In contrast, the maximal survival rate for spore-forming *B. subtilis* bacteria (7109–9804%) and *C. albicans* yeast (138–1169%) was achieved relatively slowly, only after 72 h. Finally, *A. niger* moulds achieved maximal survival rate the slowest (354–429% after 72 and 96 h).

Statistical analysis of bacteria number for the same incubation times showed that nonwovens A (PP/PEL, needle-punched nonwoven), C (PPQ, corona charged melt-blown nonwoven) and D (PP, spun-bonded nonwoven) favoured growth the most. *B. subtilis* bacteria (after 72 h incubation) grew the least on nonwoven B (PET, needle-punched nonwoven). For *S. aureus* (after 8 h incubation) and *E. coli* (after 72 and 96 h) bacteria, reduced survival on nonwoven E (PP, calandered needle-punched nonwoven) was noted. In comparison to the other nonwovens tested, we found that nonwoven B was least favourable for *C. albicans* yeast growth. The decrease in survival rate from 108% (after 4 h of incubation) and to 41% (after 96 h) indicates a gradual dying of yeast on this nonwoven. The difference in the survival rate of *A. niger* was not statistically significant for all nonwovens tested.

### 3.2. Nonwovens Structural Parameters and Contact Angle

The results of mass per unit area, thickness and contact angle measurements for the nonwovens are presented in Table 3.

**Figure 2:**
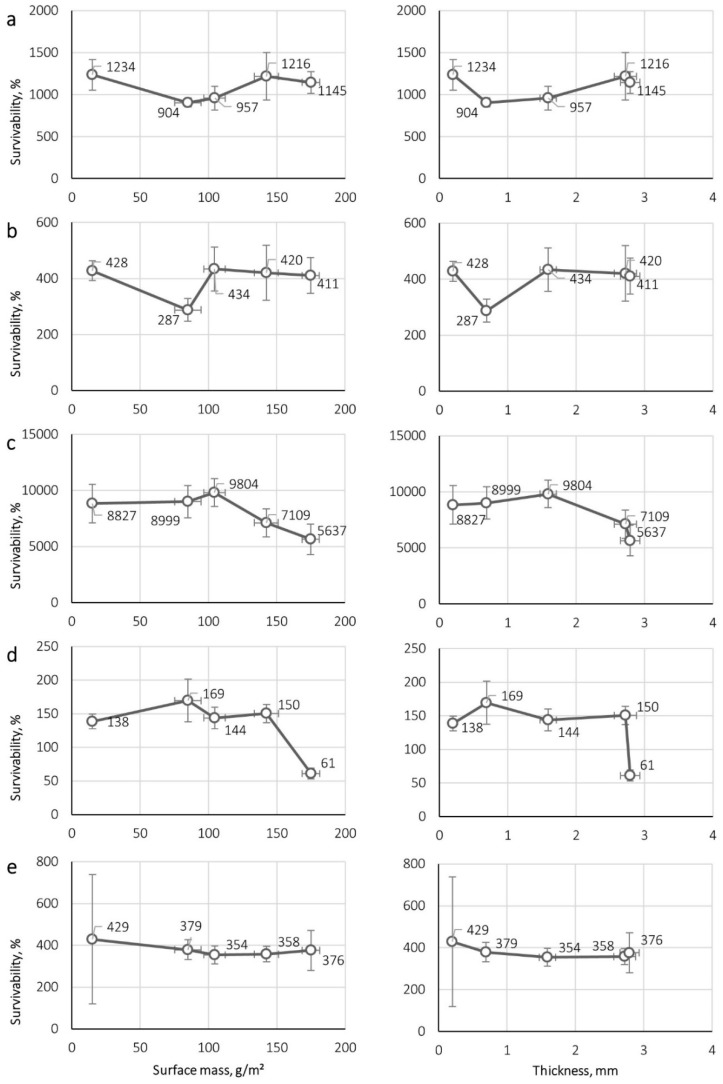
Dependence of the survivability of microorganisms on nonwovens on the mass per unit area (left) and the thickness of nonwoven (right) for (**a**) *E. coli*, (**b**) *S. aureus*, (**c**) *B. subtilis*, (**d**) *C. albicans*, (**e**) *A. niger*.

**Figure 3:**
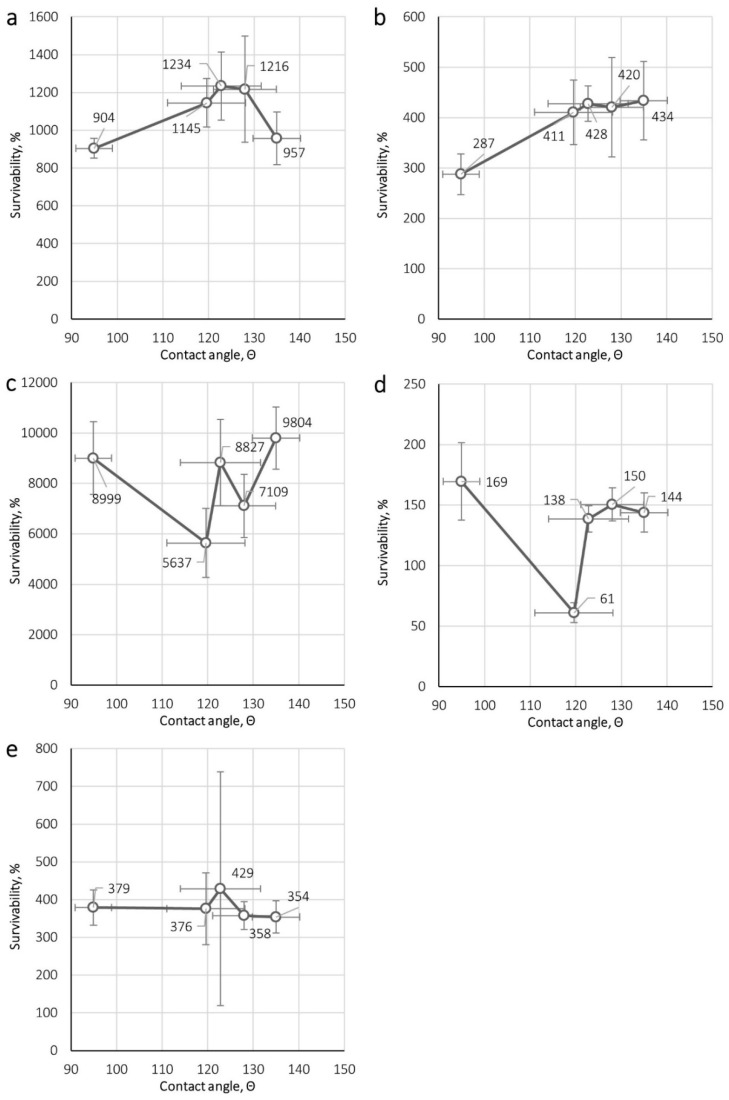
Dependence of the survivability of microorganisms on nonwoven contact angle for (**a**) *E. coli*, (**b**) *S. aureus*, (**c**) *B. subtilis*, (**d**) *C. albicans*, (**e**) *A. niger*.

Figure 2 and Figure 3 show the correlation between microorganism survival on nonwovens and their mass per unit area, thickness and contact angle. The correlations were determined for the maximal survival achieved for each microorganism, i.e., 24 h for *E. coli* and *S. aureus*, 72 h for *B. subtilis* and yeast *C. albicans*, and 96 h for *A. niger*.

A particularly important conclusion from our research is the lack of a clear relationship between the degree of fibre wettability (evaluated by the contact angle) and their structural parameters, and the tendency for the microorganism to grow on their surface. In none of the cases, was there an unequivocal relationship between the structural parameters of a nonwoven and microorganism survival (Figure 2). It should be emphasized that from the point of view of filtration efficiency of biological particles, these parameters are of utmost importance. Based on the assumption that more effective filtering materials would accumulate more particles favouring microorganism growth, we used nonwovens endowed with significantly different mass per unit area and thickness, including a highly effective electret melt-blown nonwoven, for testing. However, contrary to expectations, even in the case of nonwovens made from the same raw material (nonwovens C, D and E made of PP), both downward (for *S. aureus*, *B. subtilis* bacteria and *C. albicans* yeast) and upward (for *E. coli* bacteria and *A. niger* mould) trends in microorganism survival were observed. This property is therefore not related to the structural parameters of nonwovens, and hence to their filtration efficiency.

The contact angle measurement results (Table 3) showed that it ranged from 94 to 135, indicating that the wettability was poor for all filtering nonwovens. The correlations depicted in Figure 3 show that there were no significant differences between microorganism survival and the determined contact angle. On the other hand, the conclusions of previously published studies [18,19,20,21], indicated that the moisture content in the filtering nonwovens (the total amount of water accumulated in the fibrous structure) has a dominant influence on microorganism survival. Taking the above into consideration, it can be stated that the characteristics linked to fibre wettability (such as chemical nature of the fibre surface and the fibre geometry) are less important in the context of microbial growth than the nonwoven’s ability to accumulate moisture under real-use conditions, which would depend not only on the wettability of the nonwoven but also on the characteristics of the fibrous structure and external conditions (e.g., relative humidity or the flow pattern and velocity of the passing air). Of course, to fully support this hypothesis experiments that more adequately mimic real-use conditions should be conducted, including the studies of the influence of such factors as ambient temperature and humidity conditions, content of inorganic or organic dust as well as the presence, acidic, or alkaline sweat on the microorganism’s survival on nonwoven materials used for construction of FFRs.

Commercial FFRs used at workplaces consist mostly of filtering nonwovens described within the study. In particular, this concerns high-efficiency PP melt-blown nonwovens, which are the basic structural material of FFRs. Translating the results of laboratory tests into the actual use, it should be stated that the more effective the FFRs are, the more microorganisms will accumulate inside them. Their growth can constitute a danger to the user [21,22,23]. Therefore, the use of standard FFRs throughout the working shift for protection against biological agents does not guarantee a sufficient protection of the user. A much safer solution is the application of nonwovens with biocidal properties to the construction of FFRs [4,5,6,7,12,13]. However, such FFRs are not widely available. Another option is the frequent replacement of standard FFRs or their disinfection.

## 4. Conclusions

Microorganism growth on all filtering nonwovens utilized for FFR construction was confirmed. Their survival on pure nonwovens strongly depended on the species. The highest survival rate over short incubation time was achieved by *E. coli* and *S. aureus* bacteria. Maximal survival was achieved at later time points by spore-forming *B. subtilis* bacteria and *C. albicans* yeast, while *A. niger* moulds took the longest, i.e., after 96 hours. These differences stemmed from the growth physiology of the microorganisms studied. Microorganism survival rate is negligibly impacted by the type of filtering nonwoven, as there was no obvious relationship between survival and nonwoven characteristics (mass per unit area, thickness and contact angle). Based on our research, we also established that the composition of nonwovens did not significantly affect microorganism survival.

## Figures and Tables

**Table 1:** Characteristics of filtering nonwovens.

Notation	Raw Material Composition	Type of Nonwoven	Function in FFR
A	polypropylene/polyacrylonitrile (PP/PEL)	needle-punched nonwoven	stiffening of the FFR structure, pre-filtration of coarse dust particles
B	polyethylene (PET)		
C	polypropylene (PPQ)	corona charged melt-blown nonwoven	high-efficiency filtration of fine dust particles
D	polypropylene (PP)	spun-bonded nonwoven	pre-filtration of coarse dust particles
E	polypropylene (PP)	calandered needle-punched nonwoven	stiffening of the FFR structure,

**FFR**: filtering facepiece respirator.

**Table 2:** Characteristics of microorganisms.

Microorganisms		Species	Collection Reference Number	Inoculum Density, CFU/mL
Bacteria		*Escherichia coli*	ATCC 10536	1.12 × 10^9^ ± 4.06 × 10^8^
		*Staphylococcus aureus*	ATCC 6538	1.05 × 10^9^ ± 3.60 × 10^8^
		*Bacillus subtilis*	NCAIM 01644	6.67 × 10^8^ ± 1.06 × 10^8^
Fungi	Yeast	*Candida albicans*	ATCC 10231	1.32 × 10^8^ ± 1.93 × 10^7^
	Mould	*Aspergillus niger*	ATCC 16404	3.70 × 10^7^ ± 8.87 × 10^6^

ATCC: American Type Culture Collection; NACIM: National Collection of Agricultural and Industrial Microorganisms.

**Table 3:** Structural parameters and contact angle of nonwovens.

Type of Nonwoven	Mass per unit Area, g/m^2^	Thickness, mm	Contact Angle, Θ
A	142.4 ± 8.8	2.72 ± 0.16	128.0 ± 6.9
B	174.9 ± 6.3	2.79 ± 0.14	119.6 ± 8.6
C	104.5 ± 7.8	1.59 ± 0.12	135.0 ± 5.2
D	15.1 ± 1.5	0.20 ± 0.01	122.8 ± 8.8
E	85.0 ± 9.6	0.69 ± 0.02	94.9 ± 4.0

## Supplementary Materials

The following are available online at https://www.mdpi.com/1660-4601/16/7/1154/s1. Table S1: Number of microorganisms on the nonwovens depending on the incubation time.
