# Supplementary material for: Survival of Microorganisms on Nonwovens Used for the Construction of Filtering Facepiece Respirators
Source: Int J Environ Res Public Health. 2019 Mar 31;16(7):1154. doi: 10.3390/ijerph16071154 (PMC6480171; doi:10.3390/ijerph16071154)
Supplement: Supplementary file 1 [file ijerph-16-01154-s001.pdf]

International Journal of
Environmental Research and Public Health

MDPI

Supplementary materials: Survival of microorganisms on nonwovens used for the construction of filtering facepiece respirators

Katarzyna Majchrzycka[1], Małgorzata Okrasa[1*], Justyna Szulc[2], Anita Jachowicz[2] and Beata Gutarowska[2]

Table S1. Number of microorganisms on the nonwovens depending on the incubation time.

Time, h	Number of Microorganisms Depending on Incubation Time and Nonwoven Type, CFU/ml				
	A	B	C	D	E
			E. coli		
0	$6.2\times10^{5a}\pm2.1\times10^{5}$	$6.7\times10^{5a}\pm1.4\times10^{5}$	$6.3\times10^{5a}\pm1.3\times10^{5}$	$5.1\times10^{5a}\pm1.1\times10^{5}$	$6.4\times10^{5a}\pm1.2\times10^{5}$
4	$3.4\times10^{6*\#a}\pm6.3\times10^{5}$	$2.8\times10^{6*\#a}\pm6.3\times10^{5}$	$2.6\times10^{6*\#a}\pm9.8\times10^{5}$	$2.5\times10^{6*\#a}$v2.1×10^{5}	$2.6\times10^{6*\#a}\pm4.1\times10^{5}$
8	$6.2\times10^{6*\#a}\pm3.7\times10^{5}$	$6.4\times10^{6*\#a}\pm8.1\times10^{5}$	$5.3\times10^{6*\#ad}\pm7.2\times10^{5}$	$5.2\times10^{6*\#bcd}\pm7.9\times10^{5}$	$5.3\times10^{6*\#bcd}\pm4.7\times10^{5}$
24	$7.0\times10^{6*\#a}\pm7.4\times10^{5}$	$7.5\times10^{6*\#b}\pm1.1\times10^{6}$	$5.9\times10^{6*\#a}\pm4.9\times10^{5}$	$6.18\times10^{6*\#a}\pm1.6\times10^{6}$	$5.8\times10^{6\#a}\pm1.1\times10^{6}$
48	$4.7\times10^{6*\#a}\pm8.6\times10^{5}$	$4.8\times10^{6*\#a}\pm1.2\times10^{6}$	$3.9\times10^{6*\#a}\pm8.3\times10^{5}$	$3.6\times10^{6*\#a}\pm3.8\times10^{5}$	$3.9\times10^{6*\#a}\pm9.1\times10^{5}$
72	$4.8\times10^{6\#a}\pm7.8\times10^{5}$	$4.5\times10^{6\#a}\pm7.8\times10^{5}$	$3.6\times10^{6\#ac}\pm3.8\times10^{5}$	$2.9\times10^{6\#bc}\pm1.5\times10^{6}$	$2.5\times10^{6*\#bc}\pm5.7\times10^{5}$
96	$3.4\times10^{6*\#a}\pm3.1\times10^{5}$	$3.6\times10^{6*\#a}\pm4.0\times10^{5}$	$3.6\times10^{6\#a}\pm8.8\times10^{5}$	$2.8\times10^{6\#a}\pm9.0\times10^{5}$	$2.5\times10^{6\#b}\pm3.7\times10^{5}$
			S. aureus		
0	$5.8\times10^{5a}\pm2.2\times10^{5}$	$5.3\times10^{5a}\pm1.5\times10^{5}$	$5.4\times10^{5a}\pm1.4\times10^{5}$	$5.7\times10^{5a}\pm1.4\times10^{5}$	$5.8\times10^{5a}\pm1.8\times10^{5}$
4	$6.9\times10^{5a}\pm2.0\times10^{5}$	$7.1\times10^{5*\#a}\pm2.6\times10^{4}$	$7.3\times10^{5*\#a}\pm1.7\times10^{5}$	$6.8\times10^{5a}\pm1.7\times10^{5}$	$7.6\times10^{5*\#a}\pm1.8\times10^{5}$
8	$2.4\times10^{6*\#ab}\pm1.3\times10^{6}$	$2.2\times10^{6*\#a}\pm6.2\times10^{5}$	$1.6\times10^{6*\#a}\pm5.0\times10^{5}$	$1.6\times10^{6*\#a}\pm3.2\times10^{5}$	$1.0\times10^{6\#ac}\pm7.1\times10^{5}$
24	$2.3\times10^{6\#a}\pm2.8\times10^{5}$	$2.1\times10^{6\#a}\pm3.9\times10^{5}$	$2.2\times10^{6*\#a}\pm2.1\times10^{5}$	$2.5\times10^{6*\#a}\pm6.3\times10^{5}$	$1.6\times10^{6*\#b}\pm2.9\times10^{5}$
48	$2.3\times10^{6\#a}\pm6.1\times10^{5}$	$2.0\times10^{6\#a}\pm3.6\times10^{5}$	$2.1\times10^{6\#a}\pm2.6\times10^{5}$	$2.2\times10^{6\#a}\pm2.6\times10^{5}$	$1.8\times10^{6\#a}\pm5.0\times10^{5}$
72	$2.1\times10^{6\#a}\pm4.47\times10^{5}$	$1.8\times10^{6\#a}\pm4.1\times10^{5}$	$1.8\times10^{6\#a}\pm4.5\times10^{5}$	$2.2\times10^{6\#a}\pm5.4\times10^{5}$	$1.7\times10^{6\#a}\pm3.9\times10^{5}$
96	$1.9\times10^{6\#ab}\pm3.4\times10^{5}$	$1.7\times10^{6\#a}\pm3.3\times10^{5}$	$1.6\times10^{6\#a}\pm2.3\times10^{5}$	$1.6\times10^{6*\#a}\pm2.8\times10^{5}$	$1.5\times10^{6\#ac}\pm1.7\times10^{5}$
			B. subtilis		
0	$2.0\times10^{4a}\pm6.3\times10^{3}$	$1.5\times10^{4a}\pm5.8\times10^{3}$	$1.5\times10^{4a}\pm4.6\times10^{3}$	$1.7\times10^{4a}\pm3.7\times10^{3}$	$1.5\times10^{4a}\pm2.2\times10^{3}$
4	$2.0\times10^{4a}\pm3.77\times10^{3}$	$1.8\times10^{4a}\pm4.0\times10^{3}$	$1.9\times10^{4a}\pm5.8\times10^{3}$	$1.8\times10^{4a}\pm5.7\times10^{3}$	$1.9\times10^{4*\#a}\pm5.0\times10^{3}$
8	$2.9\times10^{4*\#a}\pm4.1\times10^{3}$	$2.9\times10^{4*\#a}\pm9.0\times10^{3}$	$2.9\times10^{4\#a}\pm1.8\times10^{4}$	$2.9\times10^{4*\#a}\pm3.6\times10^{3}$	$2.9\times10^{4*\#a}\pm6.2\times10^{3}$
24	$7.0\times10^{5*\#a}\pm6.7\times10^{4}$	$6.8\times10^{5*\#a}\pm5.1\times10^{4}$	$6.6\times10^{5*\#a}\pm5.5\times10^{4}$	$6.5\times10^{5*\#a}\pm7.5\times10^{4}$	$6.6\times10^{5*\#a}\pm7.4\times10^{4}$
48	$1.3\times10^{6*\#a}\pm1.5\times10^{5}$	$1.1\times10^{6*a}\pm1.7\times10^{5}$	$1.2\times10^{6*\#a}\pm1.0\times10^{5}$	$1.3\times10^{6*\#a}\pm1.7\times10^{5}$	$1.3\times10^{6*\#a}\pm3.8\times10^{5}$
72	$1.3\times10^{6\#a}\pm2.6\times10^{5}$	$8.0\times10^{5*\#b}\pm8.7\times10^{4}$	$1.4\times10^{6\#a}\pm4.3\times10^{5}$	$1.4\times10^{6*\#a}\pm6.7\times10^{4}$	$1.4\times10^{6\#a}\pm3.8\times10^{5}$
96	$7.8\times10^{5*\#a}\pm1.6\times10^{5}$	$5.9\times10^{5*\#ab}\pm9.5\times10^{4}$	$7.5\times10^{5*\#a}\pm1.1\times10^{5}$	$7.9\times10^{5*\#ac}\pm7.6\times10^{4}$	$7.1\times10^{5*\#a}\pm2.2\times10^{5}$
			C. albicans		
0	$3.7\times10^{4a}\pm4.8\times10^{3}$	$3.4\times10^{4a}\pm8.2\times10^{3}$	$3.9\times10^{4a}\pm8.8\times10^{3}$	$3.6\times10^{4a}\pm5.9\times10^{3}$	$3.0\times10^{4a}\pm8.3\times10^{3}$
4	$4.3\times10^{4a}\pm7.3\times10^{3}$	$3.6\times10^{4a}\pm6.7\times10^{3}$	$4.2\times10^{4a}\pm1.1\times10^{4}$	$3.7\times10^{4a}\pm1.8\times10^{4}$	$3.2\times10^{4a}\pm6.4\times10^{3}$
8	$4.3\times10^{4\#a}\pm4.3\times10^{3}$	$3.5\times10^{4a}\pm1.1\times10^{4}$	$4.3\times10^{4a}\pm1.2\times10^{4}$	$3.8\times10^{4a}\pm1.6\times10^{4}$	$3.4\times10^{4a}\pm5.4\times10^{3}$
24	$4.6\times10^{4\#ab}\pm5.5\times10^{3}$	$3.4\times10^{4ac}\pm5.8\times10^{3}$	$4.5\times10^{4ad}\pm9.1\times10^{3}$	$4.0\times10^{4a}\pm7.3\times10^{3}$	$4.0\times10^{4\#a}\pm9.9\times10^{3}$
48	$4.9\times10^{4\#}\pm3.7\times10^{3}$	$2.8\times10^{4*\#}\pm5.9\times10^{3}$	$4.8\times10^{4}\pm1.1\times10^{4}$	$4.3\times10^{4\#}\pm7.3\times10^{3}$	$4.2\times10^{4\#}\pm8.2\times10^{3}$
72	$5.5\times10^{4*\#a}\pm3.0\times10^{3}$	$2.0\times10^{4*\#b}\pm3.0\times10^{3}$	$5.5\times10^{4\#a}\pm7.9\times10^{3}$	$4.9\times10^{4\#a}\pm6.5\times10^{3}$	$4.9\times10^{4*\#a}\pm5.1\times10^{3}$
96	$5.4\times10^{4\#a}\pm6.4\times10^{3}$	$1.4\times10^{4*\#b}\pm4.0\times10^{3}$	$5.3\times10^{4\#a}\pm7.4\times10^{3}$	$4.74\times10^{4\#a}\pm7.3\times10^{3}$	$4.5\times10^{4\#a}\pm2.0\times10^{4}$
			A. niger		
0	$1.8\times10^{4a}\pm4.8\times10^{3}$	$1.8\times10^{4a}\pm6.4\times10^{3}$	$1.8\times10^{4a}\pm5.5\times10^{3}$	$1.7\times10^{4a}\pm9.1\times10^{3}$	$1.8\times10^{4a}\pm6.8\times10^{3}$

4	$2.2\times10^{4a}\pm8.3\times10^{3}$	$1.9\times10^{4a}\pm1.8\times10^{3}$	$1.9\times10^{4a}\pm5.2\times10^{3}$	$1.9\times10^{4a}\pm7.0\times10^{3}$	$1.9\times10^{4a}\pm1.3\times10^{4}$
8	$2.6\times10^{4\#a}\pm9.6\times10^{3}$	$2.2\times10^{4a}\pm6.6\times10^{3}$	$1.9\times10^{4a}\pm3.0\times10^{3}$	$2.1\times10^{4a}\pm5.7\times10^{3}$	$1.9\times10^{4a}\pm2.7\times10^{3}$
24	$9.0\times10^{3*\#a}\pm8.3\times10^{3}$	$5.6\times10^{3*\#a}\pm4.8\times10^{3}$	$6.7\times10^{3*\#a}\pm2.8\times10^{3}$	$5.4\times10^{3*\#a}\pm3.6\times10^{3}$	$5.0\times10^{3*\#a}\pm4.1\times10^{3}$
48	$3.1\times10^{4*\#a}\pm5.6\times10^{3}$	$2.4\times10^{4*a}\pm8.6\times10^{3}$	$2.6\times10^{4*\#a}\pm6.5\times10^{3}$	$2.9\times10^{4*\#a}\pm6.4\times10^{3}$	$2.6\times10^{4*\#a}\pm8.7\times10^{3}$
72	$5.8\times10^{4*\#a}\pm1.1\times10^{4}$	$5.8\times10^{4*\#a}\pm8.0\times10^{3}$	$5.4\times10^{4*\#a}\pm1.7\times10^{4}$	$5.5\times10^{4*\#a}\pm6.2\times10^{3}$	$5.8\times10^{4*\#a}\pm6.3\times10^{3}$
96	$6.4\times10^{4\#a}\pm1.4\times10^{4}$	$6.3\times10^{4\#a}\pm1.0\times10^{4}$	$6.3\times10^{4\#a}\pm1.4\times10^{4}$	$6.6\times10^{4*\#a}\pm9.8\times10^{3}$	$6.6\times10^{4\#a}\pm1.7\times10^{4}$

* - statistically significant differences between microorganism number after subsequent incubation times (ANOVA, α=0.05, Tukey, α=0.05); # - statistically significant differences between microorganism number after a given incubation time and after t = 0 h (ANOVA, α=0.05, Tukey, α=0.05); [a], [b], [c], [d] - statistically significant differences between microorganism number on different nonwovens (ANOVA, α = 0.05, Tukey, α = 0.05). ANOVA: one-way analysis of variance.
